# Bluetongue and Epizootic Haemorrhagic Disease virus in local breeds of cattle in Kenya

**DOI:** 10.1016/j.rvsc.2012.11.001

**Published:** 2013-06

**Authors:** P.G. Toye, C.A. Batten, H. Kiara, M.R. Henstock, L. Edwards, S. Thumbi, E.J. Poole, I.G. Handel, B.M.deC. Bronsvoort, O. Hanotte, J.A.W. Coetzer, M.E.J. Woolhouse, C.A.L. Oura

**Affiliations:** a The Pirbright Institute, Ash Road, Pirbright, Woking, Surrey GU24 0NF, UK; b The International Livestock Research Institute, P.O. Box 30709, Nairobi 00100, Kenya; c Centre for Immunology, Infection & Evolution, University of Edinburgh, Edinburgh EH9 3JT, UK; d The Roslin Institute, University of Edinburgh, Easter Bush EH25 9RG, UK; e School of Biology, University of Nottingham, Nottingham NG7 2RD, UK; f Department of Veterinary Tropical Diseases, Faculty of Veterinary Science, University of Pretoria, Private Bag X04, Onderstepoort 0110, South Africa; g School of Veterinary Medicine, University of the West Indies, Trinidad and Tobago

**Keywords:** Bluetongue, Epizootic Haemorrhagic Disease, Kenya, Prevalence

## Abstract

The presence of bluetongue virus (BTV) and Epizootic Haemorrhagic Disease virus (EHDV) in indigenous calves in western Kenya was investigated. Serum was analysed for BTV and EHDV antibodies. The population seroprevalences for BTV and EHDV for calves at 51 weeks of age were estimated to be 0.942 (95% CI 0.902–0.970) and 0.637 (95% CI 0.562–0.710), respectively, indicating high levels of circulating BTV and EHDV. The odds ratio of being positive for BTV if EHDV positive was estimated to be 2.57 (95% CI 1.37–4.76). When 99 calves were tested for BTV and EHDV RNA by real-time RT-PCR, 88.9% and 63.6% were positive, respectively. Comparison of the serology and real-time RT-PCR results revealed an unexpectedly large number of calves that were negative by serology but positive by real-time RT-PCR for EHDV. Eight samples positive for BTV RNA were serotyped using 24 serotype-specific real-time RT-PCR assays. Nine BTV serotypes were detected, indicating that the cattle were infected with a heterogeneous population of BTVs. The results show that BTV and EHDV are highly prevalent, with cattle being infected from an early age.

## 1 Introduction

Bluetongue virus (BTV) and Epizootic Haemorrhagic Disease virus (EHDV) are members of the genus Orbivirus, family Reoviridae (Mertens et al., 2005) and are transmitted by biting midges (*Culicoides* spp.). Bluetongue (BT) was first described in Africa in the early 19th century and can infect all species of ruminants, although clinical outbreaks are usually seen in susceptible European sheep breeds. There have been multiple incursions of BTV into Europe from Africa, the most serious caused by the strain of BTV serotype 8 (BTV-8). The outbreak spread across Europe between 2006 and 2009 and caused clinical signs in cattle, goats and sheep (EFSA Panel on Animal Health and Welfare, 2011). As limited sequence information is available for BTV strains circulating across Africa, it was not possible to conclude with certainty the origin of this virus, however full genome sequence analysis indicated that it may have originated from sub-Saharan Africa (Maan et al., 2008).

EHDV primarily infects deer, and cattle are thought to act as a reservoir. Outbreaks were reported in Morocco and Israel in 2006 and Turkey in 2007, where cattle exhibited mild clinical signs (Temizel et al., 2009; Yadin et al., 2008). Very little is known about the distribution of EHDV in Africa apart from the fact that EHDV-3 (now reclassified as EHDV-1) and EHDV-4 were isolated in Nigeria in the late 1960s and EHDV (serotype unknown) was isolated in South Africa in the 1990s (Savini et al., 2011).

The aim of this study was to improve current knowledge of the prevalence and distribution of EHDV and BTV in domestic cattle in sub-Saharan Africa. The study set out to estimate the seroprevalence of EHDV and BTV antibodies and the prevalence of infection (through the detection of viral RNA) and to identify the BTV and EHDV serotypes in a subset of samples from cattle in western Kenya.

## 2 Materials and methods

### 2.1 Study site

**Fig. 1:**
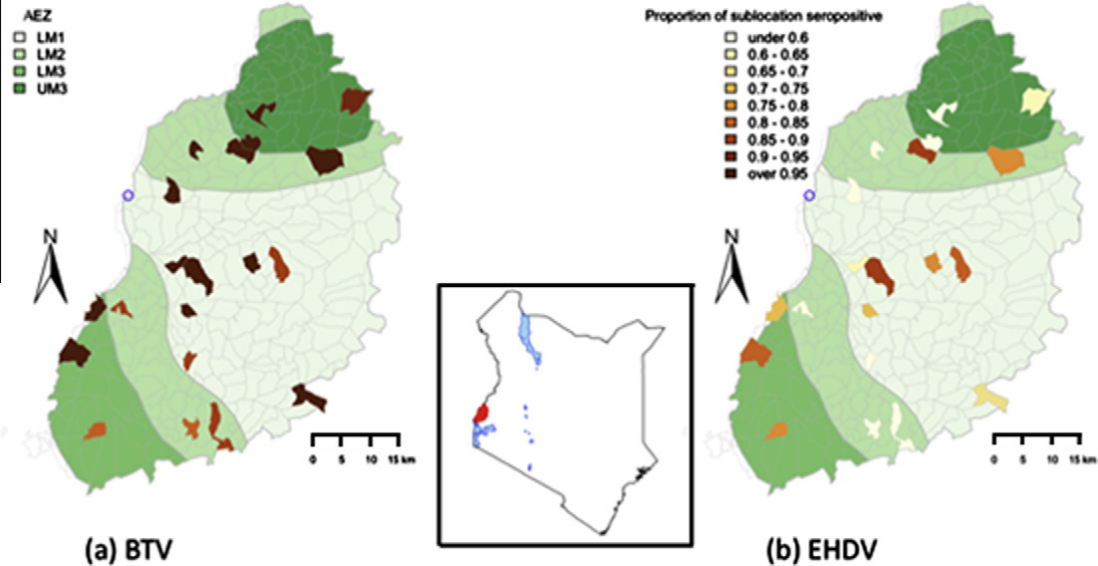
Map of the study area showing the AEZs within the study area and the 20 sublocations from which calves were recruited. The distribution of calves seropositive at 51 weeks for BTV (a) and EHDV (b) is also shown. The inset map shows the location of the study area in western Kenya, and the circle indicates the location of the project laboratory in Busia.

The samples analysed in this study were collected as part of the ‘IDEAL’ (Infectious Diseases in East African Livestock) project, which monitored infections in 548 indigenous calves, from birth to death or 12 months of age, in western Kenya, and is described in detail by Bronsvoort et al. (submitted). The field component of the study was carried out between October 2007 and September 2010, and the calves were located in households within 45 km of the town of Busia on the Kenya/Uganda border. The study area (Fig. 1) stretches from Lake Victoria in the south–west to the slopes of Mt. Elgon in the north–east and encompasses four Agro-Ecological Zones (AEZ): Lower Midlands (LM) 1, LM2, LM3 and Upper Midlands 3 (Jaetzold and Schimdt, 1983). The area has a warm and moist tropical climate with a bimodal rainfall pattern with two peaks (March to May and October to December), although there is moderate rainfall throughout year. Most of the area is cultivated but interspersed with wetlands covered with grassland and often used for communal grazing. The chief farming system is a small holder mixed crop/livestock system and the predominant breed of cattle is the small East African Zebu. Farmers also keep other livestock especially sheep and poultry. The calf selection was stratified by sublocation, which is the smallest administrative unit in Kenya, with the aim of recruiting the same number of calves per sublocation.

Calves were recruited during the first week of age, usually within the first 3–7 days after birth, and were routinely visited every 5 weeks until death or 51 weeks of age. Calf locations were geo-referenced using hand-held GPS devices (Garmin 12, Garmin Kansas, USA). The 5-week interval was chosen for logistical reasons to allow the field study to be completed within 3 years. The study was designed so that calf recruitment by sublocation was evenly distributed throughout the year. There is no planned breeding programme in these farms, with most animals being sired by an available bull. Some farmers made efforts to take their cows to specific bulls in the neighbourhood. The cattle grazed in communal grazing fields or were tethered in the household compound. Households which used stall-feeding were excluded from the study.

### 2.2 Sampling

At each 5-week visit the calves were clinically examined and biological samples were obtained for laboratory analysis. The samples used in this study were final visit samples, which were obtained from the calves at the last routine visit prior to the calf leaving the study or reaching 51 weeks of age. For serology, jugular vein blood was collected into plain Vacutainer™ (Becton Dickinson) tubes. After clotting, serum was recovered and aliquots were stored at −20 °C until use. Blood for RNA extraction was collected into EDTA-Vacutainer™ tubes, aliquotted into cryovials and stored at −20 °C. The samples were processed on the day of collection in a laboratory established by the project in Busia.

### 2.3 Serology

The detection of BTV-specific antibodies in serum was performed using a sandwich (double antigen) ELISA assay (ID-Screen Bluetongue Early detection ELISA, ID-Vet, France) according to the manufacturer’s instructions. A blocking ELISA (LSIVET EHDV BLOCKING, Laboratoire Service International, Lyon, France) for the detection of EHDV-specific antibodies was used to test each sample. The assay was performed and analysed following the manufacturer’s instructions.

### 2.4 Molecular analyses

#### 2.4.1 RNA extraction

RNA was extracted from EDTA blood samples and known BTV and EHDV positive control samples using the Universal (Qiagen, Crawley, UK) extraction robot using the ‘One for all’ protocol.

#### 2.4.2 Real-time RT-PCR

BTV RNA was detected by real-time RT-PCR using a modified version of a previously published protocol (Shaw et al., 2007), while EHDV RNA was detected using an “in house” EHDV specific real-time RT-PCR targeting genome segment 9 (unpublished).

### 2.5 Serotyping

BTV RNA was serotyped using 24 individual serotype-specific (segment 2) real-time RT-PCR assays (Mertens et al manuscript in preparation), while EHDV RNA was serotyped using 7 serotype specific gel-based RT-PCRs as described previously (Maan et al., 2010).

### 2.6 Statistical analysis

For all the statistical analysis of serological results the inconclusive results were grouped as negative to avoid overestimating the seroprevalences. The overall seroprevalences for calves at 51 weeks were estimated using a weighted adjustment for the number of breed dams in each sublocation using the R survey package (Lumley, 2011; Lumley, 2004). The sublocation specific seroprevalences were mapped using the R software version 2.9.1 (http://cran.r-project.org/) (Packages ‘Sp’, ‘classInt’, ‘RColorBrewer’ and ‘maptools’). Mean seroprevalences for each sublocation were mapped to an appropriate interval on the colour scale (Fig. 1).

A likelihood ratio test was performed to determine if the mean seroprevalence varied significantly across sublocations and was compared to a chi-squared distribution with 1 d.f. The association between the seroprevalences for these two infections was estimated using a weighted hierarchical logistic model using the *svyglm* command in the R survey package (Lumley, 2011; Lumley, 2004).

**Fig. 2:**
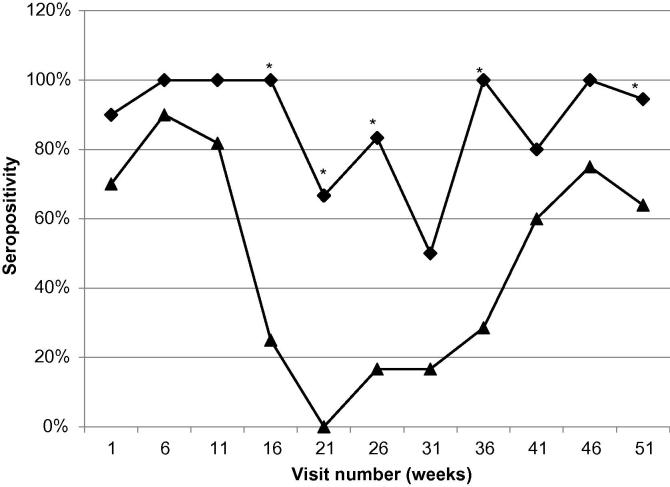
Percentage of calves, by time of visit, that were seropositive for BTV (♦) and EHDV (▴). (^∗^) Indicates the visit times for which the percentages for the two diseases were significantly different at the 5% level of significance (*p* < 0.05).

A 2-sample normal approximation binomial test was used to compare the percentage of positive BTV and EHTV results distributed by age at sampling (Fig. 2).

## 3 Results

### 3.1 Clinical disease

The calves in the study population were given a comprehensive clinical examination at each routine 5-week visit. The calves were also examined by the field team if a clinical episode was reported by the owner or local animal health worker between the 5-week visits. No overt clinical signs indicating infection with BTV or EHDV were reported throughout the study period.

### 3.2 Serology

The final visit serum samples from the 455 calves that survived until at least 51 weeks were analysed for BTV and EHDV antibodies and the results used to estimate the seroprevalence in the calf population in the study area. The results were adjusted to allow for the sublocation cluster effect and weighted by the number of dams in the sublocation. The seroprevalences of BTV and EHDV were estimated to be 0.942 (95% CI 0.902–0.970) and 0.637 (95% CI 0.562–0.710), respectively. The results indicate that calves in this region are exposed to a very high risk of infection from BTV, with a lesser but still considerable risk of EHDV infection. Of these calves, 281 (61.8%) were positive for both BTV and EHDV antibodies and only 14 (3.1%) were seronegative for both viruses. The odds ratio of being positive for BTV if EHDV positive was estimated to be 2.57 (95% CI 1.37–4.76).

The serology results from the final visit samples of 545 animals of the IDEAL cohort (the 455 final visit samples from the calves that survived to 51 weeks plus 90 samples from calves that died or left the study before 51 weeks) were analysed according to the age at which the calves were sampled. The results (Fig. 2) revealed different infection patterns for the two viruses. The EHDV curve reflects the loss of colostral antibody up to week 21, after which time the number of positive calves increased due to new infections. The BTV curve however does not show the same decline, which suggests that calves were infected with BTV at a younger age. The BTV results also show that over 90% of the calves took colostrum, which is in line with other observations (Toye et al., in preparation).

The serology results were also analysed to determine if the viruses were more prevalent in particular regions of the study site. As shown in Fig. 1, there did not appear to be a higher density of infection in any particular region.

### 3.3 PCR results

A total of 99 cattle (97 randomly selected from those aged 51 weeks, and one each aged 21 and 41 weeks) were tested for the presence of BTV and EHDV RNA by group-specific real-time RT-PCR. Eighty-eight animals (88.9%) were positive for BTV RNA, with cycle threshold (Ct) values ranging from 25 to 38. A total of 28 of the positive animals (31.8%) had Ct values less than 30, which indicates high levels of viraemia as found in the early stages of infection. For EHDV, 63 animals (63.6%) were positive, with Ct values ranging from 26 to 38. Of the 63 positive calves, 14 (22.2%) had Ct values less than 30. The real-time RT-PCR results also showed that of the 99 calves, 58 (58.6%) were positive for both viruses, indicating a high level of co-infection. There did not appear to be a bias towards any particular month of sampling for either virus (results not shown).

### 3.4 Comparison of serology and PCR results

Results obtained with both real-time RT-PCR and ELISA for BTV and EHDV were compared in 97 animals for which both sets of results were available (Table 1). For BTV, most of the calves were positive by both methods, with only three calves both seronegative and real-time RT-PCR negative. The results for EHDV showed that a surprisingly high number of the calves (18% or 28.6%) that were positive by real-time RT-PCR had no detectable antibody response. There was a small number of animals, 8 (8.2%) and 6 (6.2%) for BTV and EHDV, respectively, which were positive by serology but negative by real-time RT-PCR, suggesting that only these animals had resolved the viral infections.

### 3.5 Serotyping

A subset of eight samples, which had high levels of BTV RNA as indicated by their Ct values (between 25.4–29.6) in the group-specific real-time RT-PCR, was chosen for serotyping using 24 individual serotype-specific (segment 2) real-time RT-PCR assays (Mertens et al manuscript in preparation). The serotype-specific real-time RT-PCR assays are known to be less sensitive than the group-specific BTV real-time RT-PCR assay and hence only the samples with high viral loads were chosen for serotyping. The samples were from eight calves from eight different sublocations distributed evenly across the study site. In the eight samples, a total of nine BTV serotypes were detected, indicating that the cattle were infected with a heterogeneous population of BTV serotypes. BTV-7, 15, 16 and 19 were identified as single infections in four calves. Three other animals were dually infected, one with BTV-1 and 12, a second with BTV-3 and 24 and a third with BTV-22 and 24. The animal with BTV-1 and 12 had been sampled at 21 weeks of age. It was not possible to identify the serotype in the remaining animal despite its having a Ct value of 27.9 in the group-specific real-time RT-PCR. Attempts to isolate live virus from any of the samples were unsuccessful.

In two strongly positive EHDV samples (Ct values 28.6 and 26.8) it was not possible to identify the EHDV serotype present using 7 serotype specific gel-based RT-PCRs. This may have been due to a lower sensitivity of the gel-based serotyping assays compared to the real-time group-specific RT-PCR, or to mismatches in the VP-2 primer sequences which were designed from the reference EHDV strains. As for BTV, it was not possible to isolate virus from any of the samples.

## 4 Discussion

The results presented here show that BTV and EHDV are highly prevalent in this study site, and is the first study reporting the presence of EHDV antibodies and virus (viral RNA) in cattle from the East African region. Although these viruses did not seem to cause overt clinical signs in cattle, further work under the IDEAL project is investigating the clinical and sub-clinical significance of these and other pathogens.

The serology results provided the initial indication of the high prevalence of the viruses, with cattle being at substantial risk of infection from both viruses, but especially BTV. The cattle appeared to be infected from an early age and an analysis of the prevalence by sublocation indicated that the viruses were widespread throughout the study area and were not restricted to any particular AEZ. The high numbers of real-time RT-PCR positive results from samples collected from calves at 51 weeks of age suggested that there was considerable viral persistence within this age group of animals. This is supported by the very low numbers of animals which were antibody positive but real-time RT-PCR negative. It appears that a high proportion of infected cattle are likely to act as reservoirs for BTV and EHDV and thus pose a significant risk to naïve ruminants.

A comparison of the results obtained by serology and real-time RT-PCR assays for EHDV highlighted an unexpectedly large number of cattle which had no detectable antibodies but did contain circulating viral RNA. Available evidence indicates that the EHDV antibody response is long-lived. For example, EHDV antibody positive cattle are still reported from Morocco and Israel, despite no virus circulation since 2006 and 2007 respectively (El Harrack and Brenner personal communications). It is likely that EHDV antibodies persist for a similar period to BTV antibodies, which is thought to be lifelong (Ward and Carpenter, 1997). Thus, the most likely explanation is that these animals are in the early stages of infection, when there is circulating virus but no detectable antibody production. An alternative explanation, that the C-ELISA does not detect antibodies in some infected animals due to mutation in the epitope of the VP7 antigen recognised by the monoclonal antibody used to inhibit bovine antibodies in the assay, is possible but unlikely due to the highly conserved nature of this antigen (Mecham et al., 2003).

It is clear that several serotypes of BTV are circulating in the relatively small area comprising the study site. Work carried out in the 1970s reported that 19 serotypes of BTV were circulating in Kenya (Davies, 1978). However these studies were carried out using a serological assay (neutralisation tests) the results of which, due to cross-reactions between the serotypes, are very difficult to interpret in areas where multiple serotypes are circulating. Molecular tests (species-specific PCR), as used in the current study, are considered to be a more accurate way of identifying circulating serotypes, as this technique will definitively identify the viral RNA from each of the 24 BTV serotypes with no cross-reactions.

Three cattle were concurrently infected with two different serotypes of BTV, which raises the possibility of genetic exchange (reassortment) and the emergence of new, possibly highly virulent, BTV strains that could threaten both domestic and native ruminants. With the expected increase in distribution of *Culicoides*-borne viruses as the climate warms and the range of the *Culicoides* vector spreads, these viruses may pose an increased threat to naïve ruminant populations in BTV- and EHDV-free areas, particularly in North Africa and Europe, as was seen with the recent incursion of BTV-8 into northern and western Europe.

Despite repeated attempts, no live BT or EHD virus was isolated from the samples, possibly due to the storage of the blood in EDTA at −20 °C, which is not optimal for virus survival. The availability of live virus facilitates whole genome sequencing which allows for a more comprehensive comparison of the viral serotypes circulating in the study site with those from other regions.

In summary, this study reveals that cattle in Kenya are being infected from an early age with both BTV and EHDV, and that multiple serotypes of BTV are circulating in the region. Further studies, including virus isolation and genome sequencing of the isolated viruses, are required in order to understand more about the identity and nature of the circulating viruses. Understanding more about the molecular epidemiology of these viruses at source in Africa could help to identify transmission routes, which could prevent future incursions of BTV and EHDV into disease-free countries.

## Conflict of interest

There are no conflicts of interest.

## Role of the funding source

The IDEAL project was generously funded by the Wellcome Trust (Project 079445). The funders were not involved in the study design, in the collection, analysis and interpretation of the data, in the writing of the manuscript or in the decision to submit the manuscript for publication.

## Figures and Tables

**Table 1:** Comparison of results obtained by the serology and real-time RT-PCR assays for BTV and EHDV. The figures indicate the number of animals in each category.

	Serology +	Serology −	
*BTV*			
PCR +	84	2	86
PCR −	8	3	11
	92	5	
*EHDV*			
PCR +	45	18	63
PCR −	6	28	34
	51	46	
